# High visibility in two-color above-threshold photoemission from tungsten nanotips in a coherent control scheme

**DOI:** 10.1080/09500340.2017.1281453

**Published:** 2017-01-29

**Authors:** Timo Paschen, Michael Förster, Michael Krüger, Christoph Lemell, Georg Wachter, Florian Libisch, Thomas Madlener, Joachim Burgdörfer, Peter Hommelhoff

**Affiliations:** ^a^ Department of Physics, Friedrich-Alexander-Universität Erlangen-Nürnberg (FAU), Erlangen, Germany.; ^b^ Max Planck Institute of Quantum Optics, Garching, Germany.; ^c^ Institute for Theoretical Physics, Vienna University of Technology, Vienna, Austria.; ^d^ Max Planck Institute for the Science of Light, Erlangen, Germany.

**Keywords:** Nanotip, electron emitter, two-color laser field, density functional theory

## Abstract

In this article, we present coherent control of above-threshold photoemission from a tungsten nanotip achieving nearly perfect modulation. Depending on the pulse delay between fundamental (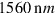) and second harmonic (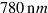) pulses of a femtosecond fiber laser at the nanotip, electron emission is significantly enhanced or depressed during temporal overlap. Electron emission is studied as a function of pulse delay, optical near-field intensities, DC bias field and final photoelectron energy. Under optimized conditions modulation amplitudes of the electron emission of 97.5% are achieved. Experimental observations are discussed in the framework of quantum-pathway interference supported by local density of states simulations.

## 1. Introduction

Field enhancement [1] at nanotips has enabled studies of non-linear and strong-field physics with moderate laser powers [2–6] and confines and enhances electron emission. Recently, the excellent transverse coherence known from DC field emission has been shown to persist in photoemission from sharp nanotips [7]. This further highlights the promise of tips as an ultrafast laser-triggered electron source of exceptional beam quality [8–11].

The mixing ratio and the relative phase of two-color laser fields augment the set of parameters to tune and to control dynamics on the (sub-)femtosecond timescale. Using such fields, above-threshold ionization of atoms [12], high-harmonic generation [13], molecular orientation [14] and polarization control of terahertz waves [15] have been investigated.

So far, two-color coherent control studies have mostly been performed with gaseous systems or macroscopic surfaces. Here, we present conclusive evidence that the localized emission of nanotips allows us to achieve nearly perfect control of electron emission yield with two-color interference. Control is achieved by variation of the phase between a 1560 nm drive pulse and a weak second harmonic admixture at 780 nm. In-situ inspection of the sample surface aids in obtaining a well-defined electron emitter to surpass the limitations of focal averaging and inhomogeneous broadening. Since the nanotip is much smaller than the focal spot sizes, it singles out local field intensities and phases, so that the inhomogeneous distribution in the laser focus does not play a detrimental role. In this contribution, we expand our findings from [16] and take advantage of the solid-state nature of the tip to investigate the influence of a strong DC bias field on the degree of control achievable in two-color photoemission by the optical phase delay. We find that large DC bias fields (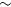 GV/m) inhibit optical control. With optimized parameters, we find a contrast of the photocurrent as function of phase delay of 97.5%.

## 2. Experimental setup

**Figure 1.**
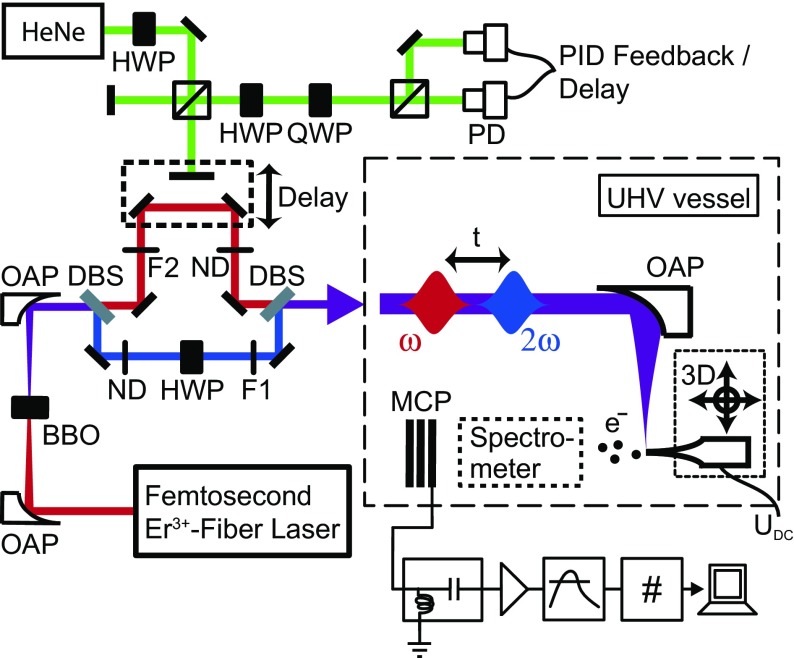
Experimental setup. Pulses from an Erbium-doped fiber laser are partially frequency-doubled in a non-linear crystal. The resulting beams are separated in a dichroic Mach-Zehnder-type interferometer to introduce time (and thereby phase) delay. Color and interference filters in both arms ensure a strict separation of fundamental and second harmonic. The delay stage can be locked to an arbitrary position using a second interferometer. Both 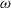 and 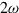 pulses are tightly focused onto the nanotip under UHV conditions by an off-axis parabolic mirror. Emitted electrons from the tip are measured using a MCP detector or a retarding-field spectrometer. Abbreviations are: OAP – off-axis parabolic mirror, BBO – beta-barium borate crystal, DBS – dichroic beam splitter, ND – neutral density filter wheel, HWP – half wave plate, QWP – quarter wave plate, F1/F2 – color and interference filters, PD – photodetector, MCP – microchannel plate detector.

Our setup is depicted in Figure 1. An amplified Erbium-doped fibre laser system emits laser pulses with a pulse length of 74 fs at a central wavelength of 1560 nm. The pulses are focused into a beta-barium borate (BBO) crystal by an off-axis parabolic mirror (OAP) for second harmonic generation to yield laser pulses with 780 nm central wavelength and a pulse length of 64 fs. Because of the parametric nature of second harmonic generation fundamental and second harmonic pulses are locked in phase. In a Mach-Zehnder-like interferometer fundamental and second harmonic beams are separated and, after manipulation, re-combined with the help of dichroic mirrors. The fundamental pulse can be delayed by virtue of apiezo-driven delay stage. Both interferometer arms contain neutral density filter wheels for intensity control; the second harmonic path is equipped with an additional waveplate for polarization adjustment. A second interferometer with a helium-neon laser is used for delay calibration and stabilization (see Figure 1, green laser path) employing *Pancharatnam’s phase* [17]. For calibration, the differential photodiode signal is recorded by a data acquisition (DaQ) card and used to determine the piezo-movement in post-processing. For active stabilization a control voltage is sent to the piezo stage from a proportional-integral (PI) feedback loop using the photodiode signal as input.

After recombination, fundamental and second harmonic pulses are tightly focused onto the nanotip inside a UHV chamber at a base pressure of 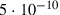 mbar using an off-axis parabolic mirror. The polarization vectors of fundamental and second harmonic light are in this contribution parallel to the tip axis, unless explicitly mentioned otherwise. The tip is mounted on a piezo-controlled 3D-translation stage and is biased with a negative voltage, resulting in a static field of up to 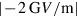 for photoemission experiments. Electron emission is detected either spatially resolved by a micro-channel plate (MCP) detector or energy-resolved using a retarding-field spectrometer. Detected events are discriminated, transformed into TTL pulses, and afterwards counted by a DaQ card. For the presented measurements in this article, the Keldysh parameter 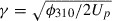 is for maximum intensities 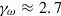 and 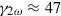. Here, 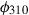 is the work function of the (310) plane of tungsten and 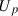 the ponderomotive energy of the electron in the respective tip-enhanced near field. For the nanotip used in the presented experiment we estimate field enhancement factors of 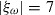 and 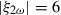.

The tungsten tips are electrochemically etched from single-crystalline wire oriented along the [310]-direction utilizing the *two-lamellae drop-off* technique [18]. Thereby the low-work-function plane (310) points in the forward direction. The tips typically display a cone-like shank that gradually reduces to a nanometer-sized apex. For further characterization of the surface of the tip apex *Field Ion Microscopy* (FIM) is applied [19] and thereby a radius of curvature of the hemispherical apex of the employed tips of about 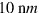 is determined.

## 3. Experimental results

**Figure 2.**
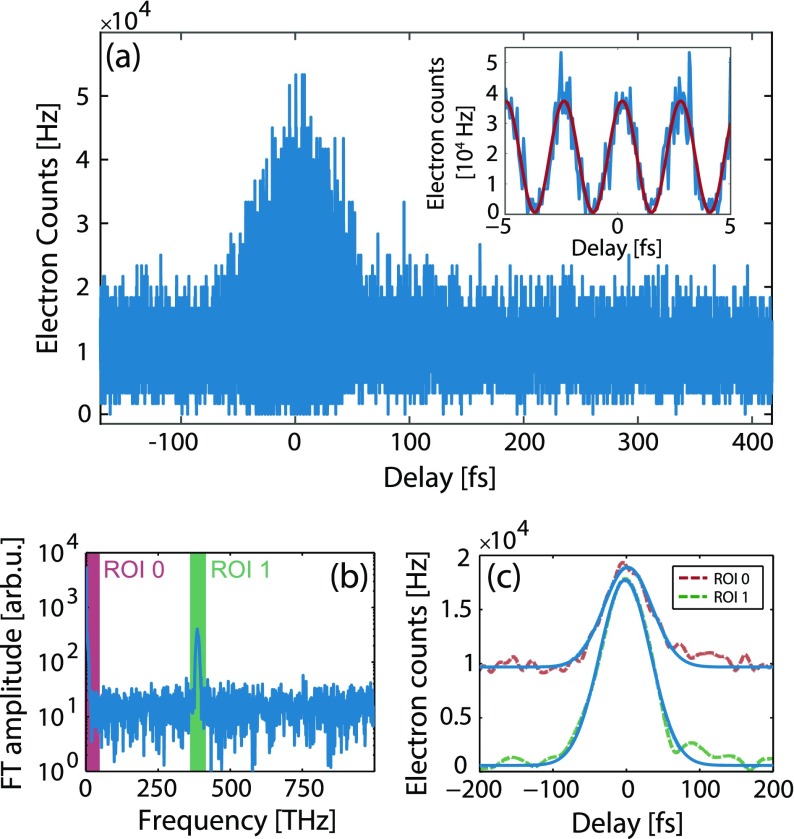
Exemplary two-color electron emission and analysis. (a) Emitted current from the tip as a function of delay of fundamental and second harmonic pulses with 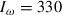 GW/cm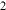, 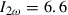 GW/cm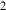 (estimated peak intensity at the tip apex) and a static field of 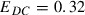 GV/m. Depending on the optical phase a dramatic increase or decrease of emission is noticeable around 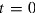. The inset shows the overlap region in more detail. A high-contrast modulation of the electron count rate is visible. A fit function (red solid line) reveals a sinusoidal behaviour and yields a visibility of 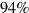. Under optimized conditions we achieved a contrast of 97.5% in a different measurement, see Figure 6. (b) Fourier transformation (FT) of the data shown in (a). Two distinct components of the signal can be extracted: A DC-part at low frequencies (highlighted in red) and a peak at 390 THz (highlighted in green). (c) Inverse-transformation of the two individual parts ROI 0 and ROI 1 and additional Hilbert transformation of data in ROI 1 (red and green dashed lines) with Gaussian fits to the data (blue solid lines).

When we vary the delay *t* between fundamental and second harmonic pulses, we observe a distinct change in electron emission from the tip when the pulses temporally overlap (see Figure 2(a)). On top of an overall increase of electron emission the current is modulated with a high contrast even for a very weak admixture of 2% second harmonic intensity. Compared to the case of non-overlapping pulses the electron emission is in this measurement increased and decreased by a factor of 3.7 and 0.12, respectively. The inset of Figure 2(a) demonstrates that the modulation can be well approximated with a sinusoidal function (red solid line). Maximum cooperative electron emission occurs when the 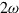 polarization vector is aligned parallel to that of the fundamental and to the tip axis. This assures optimal overlap of the near-fields induced by the two colors. Moreover, polarization vectors orthogonal to the tip axis induce high near-field regions away from the (310)plane resulting in lower emission probability. The cooperative effect is minimal for the case of perpendicular polarization vectors between 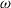 and 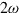 pulses, whereas the overall emission characteristics as a function of the 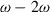 phase delay is maintained.

For quantitative investigation, we Fourier transform the measured data and obtain the dominant frequency components of the phase-dependent electron emission. The Fourier spectrum of the data of Figure 2(a) is depicted in (b). It is clearly visible that only two main frequency regions contribute, marked ROI 0 (increased average count rate, red area) and ROI 1 at a frequency of 390 THz (amplitude of modulation, green area). The variations of ROI 0 and ROI 1 as function of the delay *t* are shown in Figure 2(c). From these data, the cooperative contributions in ROI 0 and ROI 1 during temporal overlap are extracted by fitting Gaussian functions of the form(1)
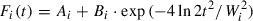



and evaluating the parameters 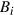. Here, 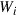 are the full widths at half maximum (FWHM) and 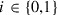.

**Figure 3.**
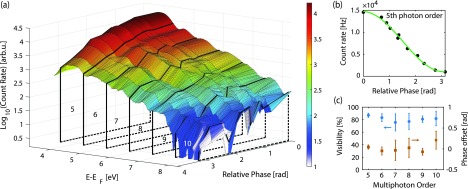
(a) Electron energy spectra for 11 different phases between 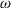 and 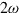 fields. Near-field intensities are 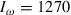 GW/cm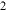 and 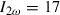 GW/cm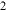 and the static field is 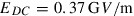. Above-threshold photon orders are indicated with numbers 5–10. An exponential drop in count rate towards higher electron energies can be seen for all phases. (b) Mean count rate of the 5th multiphoton order as function of 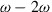 phase. Green solid line is a sinusoidal fit to the data. (c) Visibilities (blue) and phase offsets (brown) for each multiphoton order extracted from individual sinusoidal fits.

In Figure 3(a), we show the electron count rate as a function of electron energy and relative phase between the two colors. Multiphoton orders are clearly visible and the count rate drops exponentially with increasing electron energy. By changing the optical phase, the emission is homogeneously enhanced or suppressed in a sinusoidal pattern, independent of the final electron energy. For additional evaluation, the spectra are divided into individual photon orders numbered from 5 to 10. The width of the sections corresponds to the fundamental central photon energy of 0.8 eV. For each relative phase setting the mean count rate of the sections is calculated. In Figure 3(b), the results are shown exemplarily for the fifth photon order. Sinusoidal fit functions are used to evaluate this data further to obtain the visibility and phase offset with respect to maximum overall current for all multiphoton orders (see Figure 3(c)). Each visibility value agrees with the globally observed visibility of 85% for the shown data set within the error bar. The phase offset values scatter around zero, which indicates equal phases for all multiphoton orders. In [16] a more detailed analysis of the data shown in Figures 2 and 3 is presented.

**Figure 4.**
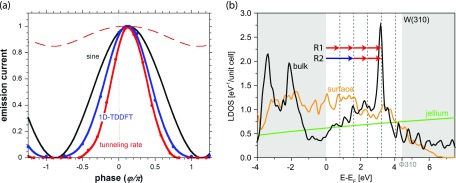
(a) Comparison of the normalized electron current in time-overlap as a function of the two-color phase delay 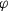 for different emission models. 1D-TDDFT simulations for a jellium (blue line) and pure tunnelling from the Fermi edge (red line) indicate an exponential current modulation compared to the experimentally observed sine-like modulation (black line). The red dashed line with small modulation depth results from tunnelling from a doorway state at an effective barrier height of 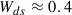 eV. (b) Ground-state LDOS simulations for tungsten in (310) direction. Simulated are surface LDOS (orange line) and bulk DOS (black line). For comparison, the DOS for a monotonically increasing jellium is shown in green. A distinct peak at 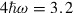 eV in the case of the bulk simulation is visible. Red and blue arrows indicate fundamental and second harmonic photon energies. On display are the two possible paths of the quantum pathway model to the bulk state.

The observed sinusoidal modulation of the photo-emitted current from the nanotip is at variance with a strong-field tunnelling model employing the rate of Yudin and Ivanov [20] which would anticipate an exponential modulation with the amplitude of the combined fields at the tip. Time-dependent density functional theory (TDDFT) calculations for a 1d-jellium model also fail to reproduce our experimental findings. In Figure 4(a), different emission mechanisms are compared. The measured pure sinusoidal modulation of the normalized electron current as a function of the two-color phase delay 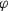 is indicated by a black line. 1D-TDDFT simulations in the jellium approximation (blue line) and pure tunnelling from the Fermi edge (red line) feature a similar visibility (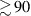
%) but narrow maxima and wide minima in contrast to the experimental results. On the other hand, tunnelling from a doorway state at an effective barrier height of 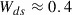 eV (red dashed line) shows only a weak modulation as a function of 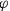 pointing to a two-pathway model as origin for the large phase contrast.

Ground-state density functional theory (DFT) simulations in 3D for tungsten reveal the reason for the discrepancy of experiment and simulation (see Figure 4(b)): the local density of states (LDOS) of tungsten is modulated considerably in the energy region between Fermi energy and vacuum level. A pronounced peak in the bulk density of states is visible at 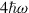 which acts as a doorway state for further emission: multiphoton electron emission can be resonantly enhanced compared to the predictions of the jellium model.

The scalings of the parameters 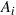 and 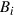 with fundamental and second harmonic intensities, the identical behaviour of electrons irrespective of their final kinetic energy and the bulk state at 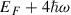 imply an emission scheme where just two pathways contribute to electron emission (see Figure 4 and [16]). From the Fermi level at least four fundamental photons (red arrows) are needed to reach the doorway state at an energy of 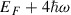. This pathway involving only fundamental photons is labeled R1. Another possible path to reach this state is shown as pathway R2. Here two fundamental photons and one additional second harmonic photon are required for the electron to be excited. Both pathways reach the same state, enabling interference due to the coherent nature of the excitation. From the excited state emission proceeds further. Emission channels incorporating more than one second harmonic photon can be neglected at small 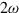 admixtures due to their very low probability.

**Figure 5.**
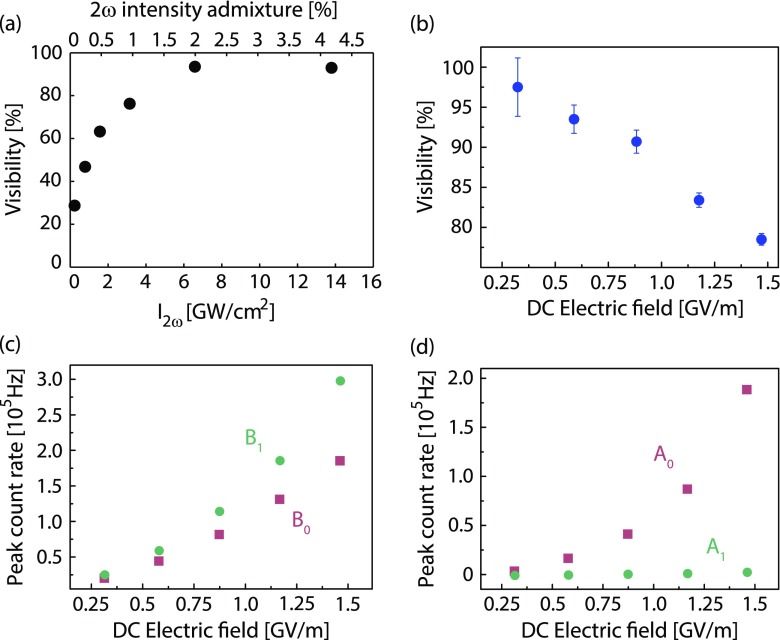
Visibility of emitted current as function of control parameters. (a) Visibility as a function of second harmonic intensity. For 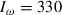 GW/cm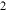 and a static field of 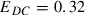 GV/m the visibility is maximum at an admixture of 2% second harmonic intensity (black circles). (b) Visibility dependence on bias tip voltage. Maximum visibility is reached for lowest tip voltage. A monotonic trend is evident. (c) and (d) Scaling of the peak amplitudes 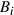 and signal offsets 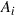 according to Equation 1 with tip bias voltage. 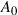 and 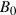 are depicted as red squares, 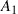 and 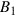 as green circles. For the data shown in (b)–(d) near-field intensities are 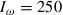 GW/cm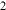 and 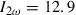 GW/cm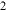.

The visibility of the modulated electron count rate is given by(2)
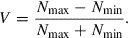



Here 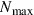 and 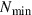 are maximum and minimum count rate determined in temporal overlap with the help of sinusoidal fits. In Figure 5(a), the visibility values for a data set with varying 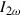-admixture, including the data of Figure 2, are shown. In this case, a 2% admixture of second harmonic leads to the highest visibility. For the data point with the highest admixture, a new frequency component at 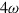 is visible in the Fourier spectrum, which indicates the onset of higher-order multiphoton processes not covered by the two-pathway model.

A further control knob for the visibility of the emission-current modulation is the tip bias voltage (Figure 5(b)–(d)). In addition to optical fields, also strong DC fields of up to 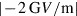 can be applied to the nanotip apex for a photoemission experiment, limited by the onset of DC field emission. Thus, for the small second harmonic intensities used in our experiment, the bias field is comparable to the maximum field of the second harmonic.

For all fundamental intensities and second harmonic admixtures we observe a monotonic decrease of the evaluated visibility with increasing magnitude of the bias voltage. This occurs despite stronger cooperative signals signified by larger fit parameters 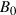 and 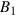 (Figure 5(c)). The decrease in visibility is mainly caused by the growth of the non-cooperative contribution to electron emission (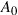, see Figure 5(d)), which grows faster with the bias field 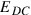 than 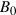 and 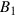. We find 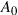 to be almost identical to and to show the same dependence on 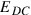 as count rates obtained by fundamental color pulses only, possibly due to increased photo-assisted field emission after one or two photon capture (note the increased surface LDOS in Figure 4 at 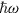 and 2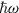) [21]. Thus, for maximum two-color phase-control of the emitted current the single-color background emission should be reduced to a minimum by decreasing the bias field applied to the tip.

**Figure 6.**
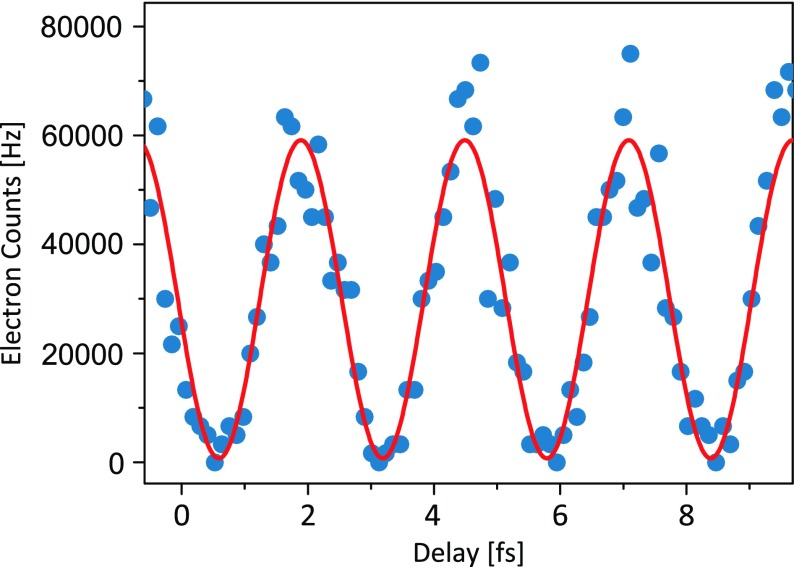
Highest achieved visibility of 97.5% in pulse overlap with intensities 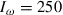 GW/cm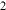, 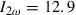 GW/cm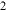 and a static field of 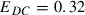 GV/m. Blue dots are measured data, the red solid line is a sinusoidal fit to the data to determine the visibility. Time 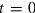 is set arbitrarily.

With optimized control parameters (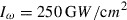, 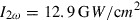, and 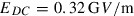) we could reach a maximum visibility of 97.5% for two-color electron emission. In Figure 6 a close-up of the modulated electron emission in the region of temporal overlap of the two colors for this maximum visibility is presented. The minimum count rate drops nearly to zero resulting in almost completely suppressed electron emission from the nanotip. Note that, while we observe that the control over the emitted current with phase delay persists and is even strengthened, for the high second harmonic intensity admixture of 5% for the measurements presented in Figures 5 and 6 the simple two-pathway model discussed above is not sufficient and has to be amended. A Fourier decomposition of the data shown in Figure 6 reveals an emerging contribution at a frequency of 768 THz (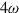). This indicates an onset of alternative emission channels. For even higher second harmonic intensities the probability for emission pathways involving more than one 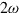 photon increases significantly. To account for this effect the pathway interference model has to be extended with higher order terms to accurately describe emission with a strong second harmonic pulse.

## 4. Outlook and conclusion

In this article we demonstrated coherently controlled two-color above-threshold photoemission from a metal nanotip. Optimizing fundamental and second harmonic intensities, as well as the static field at the tip, an optimized visibility of 97.5% was obtained, which is amongst the highest values for two-color experiments [22]. Given the solid-state nature of the nanotip the degree of control of the total electron emission is surprisingly high. A quantum pathway interference model is capable of describing the sinusoidal behaviour of electron emission as function of the optical phase between the two colors for small 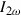 admixtures. This model is further supported by DFT calculations that indicate resonant electron emission enhancement using a doorway state at 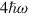.

Nanotips may in the future be used as a nanometric probe for light phases [23]. Furthermore, utilizing polarization-shaped two-color laser pulses [24] in conjunction with the polarization-sensitive near field, and the spatially varying work function at the nanotip surface [25] will allow the generation of femtosecond user-defined electron bunches.

## References

[1] Novotny L.; Hecht B. *Principles of Nano-optics*; Cambridge University Press: Cambridge, 2012.

[2] Schenk M.; Krüger M.; Hommelhoff P. *Phys. Rev. Lett.* 2010, , 257601. 21231628 10.1103/PhysRevLett.105.257601

[3] Krüger M.; Schenk M.; Hommelhoff P. *Nature* 2011, , 78–81. 10.1038/nature10196 21734706

[4] Krüger M.; Schenk M.; Hommelhoff P.; Wachter G.; Lemell C.; Burgdörfer J. *New J. Physics* 2012, , 085019.

[5] Herink G.; Solli D.; Gulde M.; Ropers C. *Nature* 2012, , 190–193. 10.1038/nature10878 22398557

[6] Piglosiewicz B.; Schmidt S.; Park D.J.; Vogelsang J.; Groß P.; Manzoni C.; Farinello P.; Cerullo G.; Lienau C. *Nat. Photonics* 2014, , 37–42.

[7] Ehberger D.; Hammer J.; Eisele M.; Krüger M.; Noe J.; Högele A.; Hommelhoff P. *Phys. Rev. Lett.* 2015, , 227601. 26196645 10.1103/PhysRevLett.114.227601

[8] Hoffrogge J.; Stein J.P.; Krüger M.; Förster M.; Hammer J.; Ehberger D.; Baum P.; Hommelhoff P. *J. Appl. Phys.* 2014, , 094506.

[9] Mueller M.; Kravtsov V.; Paarmann A.; Raschke M.B.; Ernstorfer R. *ACS Photonics* 2016, , 611–619.

[10] Vogelsang J.; Robin J.; Nagy B.J.; Dombi P.; Rosenkranz D.; Schiek M.; Groß P.; Lienau C. *Nano Lett.* 2015, , 4685–4691. 26061633 10.1021/acs.nanolett.5b01513

[11] Feist A.; Echternkamp K.E.; Schauss J.; Yalunin S.V.; Sascha S.; Ropers C. *Nature* 2015, , 200–203. 10.1038/nature14463 25971512

[12] Muller H.; Bucksbaum P.; Schumacher D.; Zavriyev A. *J. Phys. B: At., Mol. Opt. Phys.* 1990, , 2761–2769.

[13] Watanabe S.; Kondo K.; Nabekawa Y.; Sagisaka A.; Kobayashi Y. *Phys. Rev. Lett.* 1994, , 2692–2695. 10057168 10.1103/PhysRevLett.73.2692

[14] De S.; Znakovskaya I.; Ray D.; Anis F.; Johnson N.G.; Bocharova I.; Magrakvelidze M.; Esry B.; Cocke C.; Litvinyuk I.; Kling M. *Phys. Rev. Lett.* 2009, , 153002. 10.1103/PhysRevLett.103.153002 19905632

[15] Dai J.; Karpowicz N.; Zhang X.C. *Phys. Rev. Lett.* 2009, , 023001. 10.1103/PhysRevLett.103.023001 19659200

[16] Förster M.; Timo P.; Michael K.; Lemell C.; Wachter G.; Florian L.; Madlener T.; Burgdörfer J.; Hommelhoff P. *Phys. Rev. Lett.* 2016, , 217601. 10.1103/PhysRevLett.117.217601 27911540

[17] Wehner M.; Ulm M.; Wegener M. *Opt. Lett.* 1997, , 1455–1457. 18188266 10.1364/ol.22.001455

[18] Klein M.; Schwitzgebel G. *Rev. Sci. Instrum.* 1997, , 3099–3103.

[19] Müller E.W. *Adv. Electron. Electron Phys.* 1960, , 83–179.

[20] Yudin G.L.; Ivanov M.Y. *Phys. Rev. A* 2001, , 013409.

[21] Hommelhoff P.; Sortais Y.; Aghajani-Talesh A.; Kasevich M. *Phys. Rev. Lett.* 2006, , 077401. 16606139 10.1103/PhysRevLett.96.077401

[22] Ackermann P.; Scharf A.; Halfmann T. *Phys. Rev. A* 2014, , 063804.

[23] Chen C.; Elliott D. *Phys. Rev. Lett.* 1990, , 1737–1740. 10.1103/PhysRevLett.65.1737 10042350

[24] Brixner T.; Gerber G. *Opt. Lett.* 2001, , 557–559. 18040384 10.1364/ol.26.000557

[25] Yanagisawa H.; Hafner C.; Doná P.; Klöckner M.; Leuenberger D.; Greber T.; Hengsberger M.; Osterwalder J. *Phys. Rev. Lett.* 2009, , 257603. 20366286 10.1103/PhysRevLett.103.257603

